# Inhibition of the Myocardin-Related Transcription Factor Pathway Increases Efficacy of Trametinib in *NRAS*-Mutant Melanoma Cell Lines

**DOI:** 10.3390/cancers13092012

**Published:** 2021-04-22

**Authors:** Kathryn M. Appleton, Charuta C. Palsuledesai, Sean A. Misek, Maja Blake, Joseph Zagorski, Kathleen A. Gallo, Thomas S. Dexheimer, Richard R. Neubig

**Affiliations:** 1 Department of Pharmacology and Toxicology, Michigan State University, East Lansing, MI 48824, USA; kateappleton22@gmail.com (K.M.A.); charu.charuta@gmail.com (C.C.P.); blakemaj@msu.edu (M.B.); Joseph.Zagorski@helendevoschildrens.org (J.Z.); thomas.dexheimer@nih.gov (T.S.D.); 2 Department of Physiology, Michigan State University, East Lansing, MI 48824, USA; smisek@broadinstitute.org (S.A.M.); gallok@msu.edu (K.A.G.); 3 Department of Medicine, Division of Dermatology, Michigan State University, East Lansing, MI 48824, USA

**Keywords:** Rho GTPase, MEK inhibitor, resistance

## Abstract

**Simple Summary:**

Malignant melanoma is the most aggressive skin cancer, and treatment is often ineffective due to the development of resistance to targeted therapeutic agents. The most prevalent form of melanoma with a mutated *BRAF* gene has an effective treatment, but the second most common mutation in melanoma (*NRAS*) leads to tumors that lack targeted therapies. In this study, we show that *NRAS* mutant human melanoma cells that are most resistant to inhibition of the oncogenic pathway have a second activated pathway (Rho). Inhibiting that pathway at one of several points can produce more effective cell killing than inhibition of the *NRAS* pathway alone. This raises the possibility that such a combination treatment could prove effective in those melanomas that fail to respond to existing targeted therapies such as vemurafenib and trametinib.

**Abstract:**

The Ras/MEK/ERK pathway has been the primary focus of targeted therapies in melanoma; it is aberrantly activated in almost 80% of human cutaneous melanomas (≈50% *BRAF*V600 mutations and ≈30% *NRAS* mutations). While drugs targeting the MAPK pathway have yielded success in *BRAF*V600 mutant melanoma patients, such therapies have been ineffective in patients with *NRAS* mutant melanomas in part due to their cytostatic effects and primary resistance. Here, we demonstrate that increased Rho/MRTF-pathway activation correlates with high intrinsic resistance to the MEK inhibitor, trametinib, in a panel of *NRAS* mutant melanoma cell lines. A combination of trametinib with the Rho/MRTF-pathway inhibitor, CCG-222740, synergistically reduced cell viability in *NRAS* mutant melanoma cell lines in vitro. Furthermore, the combination of CCG-222740 with trametinib induced apoptosis and reduced clonogenicity in SK-Mel-147 cells, which are highly resistant to trametinib. These findings suggest a role of the Rho/MRTF-pathway in intrinsic trametinib resistance in a subset of *NRAS* mutant melanoma cell lines and highlight the therapeutic potential of concurrently targeting the Rho/MRTF-pathway and MEK in *NRAS* mutant melanomas.

## 1. Introduction

With expanding knowledge of the genomic landscape of melanoma, targeted therapies have been evolving over the last decade [1]. Almost half of the melanoma patients harbor *BRAF*^V600^ mutations, while 30% of melanomas are driven by oncogenic *NRAS* mutations [2,3]. A number of FDA-approved targeted therapies have become standard of care in *BRAF* mutant melanoma patients. Inhibitors targeting mutant *BRAF*, such as vemurafenib and dabrafenib, were initially employed as monotherapies. In recent years, *BRAF*/MEK inhibitor combinations including dabrafenib/trametinib, vemurafenib/cometinib, and encorafenib/binimetinib have been approved by the FDA, leading to median progression-free survival (PFS) of up to 14.9 months in *BRAF* mutant melanoma patients [4]. Compared to melanoma patients with *BRAF*^V600^ mutations, patients with activating *NRAS* mutations have more aggressive disease progression and poorer outcomes [5]. To date, treatment options for *NRAS* mutant melanoma patients are limited to chemotherapeutics and immunotherapies, both of which are associated with high toxicity and/or low response rates [6,7,8,9]. Unfortunately, targeted therapies for *NRAS* mutant melanoma patients are sorely lacking.

The design of specific small molecule inhibitors of oncogenic *NRAS* has been difficult, and efforts to inhibit post-translational modification of *NRAS* via farnesyltransferase inhibitors have not yet yielded approved therapies [10,11,12]. Therefore, efforts to develop targeted therapies for *NRAS* mutant melanomas have focused on signaling components downstream of *NRAS* in the MAPK pathway, such as mitogen-activated protein kinase kinase (MEK). Only a subset of patients benefits from MEK inhibitors used as single agents, and they only produce cytostatic effects, rather than cytotoxic effects, in *NRAS*-mutant melanoma cells [13]. MEK inhibitors are also associated with primary and acquired resistance as well as frequent toxicity-related adverse events [14,15]. Currently, the MEK inhibitor FCN-159, which has 10-fold higher selectivity against activated MEK1/2 compared to trametinib, is being investigated as a single agent in a phase I clinical trial (NCT03932253). It is thought that MEK inhibitors could be more effective when combined with inhibitors of other signaling mechanisms [12,15,16,17]. For example, clinical trials for the MEK inhibitor trametinib in combination with an ErbB3 antibody (NCT03580382) or a pan-RAF inhibitor, LXH254 (NCT02974725), are currently underway. However, these studies are still in early-phase clinical trials. Furthermore, primary and acquired resistance to some of the combinations, such as the MEK inhibitor MEK162 with a CDK4/6 inhibitor [18,19], are emerging. Therefore, it is critical to elucidate the mechanisms of resistance of *NRAS* mutant melanomas to MEK inhibitors and to develop new treatment strategies to overcome these clinical challenges.

In cutaneous melanoma patients, increased expression of RhoC or MRTF-A mRNA has been linked to poor overall survival [20]. The myocardin-related transcription factors (MRTF or MRTF-A and -B) are transcription cofactors acting downstream of Rho GTPases [21,22]. Rho GTPases regulate the actin cytoskeleton and have long been demonstrated to play critical roles in cellular invasion and metastasis in numerous human cancers including melanoma [23,24]. The activation of RhoA and RhoC GTPases causes the subsequent activation of an effector protein, Rho-associated protein kinase (ROCK), which leads to the formation of F-actin polymers and resultant depletion of free G-actin monomers [25]. In the cytosol, G-actin monomer binds to the RPEL domain in the N-terminal region of MRTF and sequesters MRTF in the cytosol [26]. Upon F-actin polymerization during the formation of stress fibers, MRTF translocates to the nucleus, where it cooperates with serum response factor (SRF) to induce the transcription of numerous genes involved in cell proliferation and migration [21,22]. The MRTF–SRF transcriptional axis plays a pro-metastatic role in the context of melanoma and other cancers [27]. The depletion of MRTF via RNA interference (RNAi) in the highly metastatic B16F2 melanoma cell line reduced in vitro cell migration and in vivo lung metastasis [26]. Additionally, pharmacologic inhibition of the Rho/MRTF pathway by the small-molecule CCG-203971 significantly reduced in vitro cellular migration and invasion [20,28], as well as in vivo lung metastasis in the RhoC-expressing *NRAS* mutant melanoma cell line SK-Mel-147 [20]. In addition to its anti-migratory and anti-metastatic properties, CCG-203971 induced G1-cell cycle arrest in melanoma cells. The recent observation that melanoma cells arrested in the G1 phase have higher sensitivity to MEK inhibitors [29], suggesting a potential benefit of a combination treatment with MEK inhibitors and Rho/MRTF pathway inhibitors.

Through structure–activity relationship optimization of CCG-203971, we recently reported an analog CCG-222740 with increased potency of MRTF-pathway inhibition in primary human dermal fibroblasts [30]. Additionally, CCG-222740 demonstrated a greater inhibitory effect on MRTF/SRF target genes (*ACTA2* and *CTGF*) and lesser cytotoxicity than CCG-203971 in a preclinical model of fibrosis [31]. Recent mechanistic studies with the CCG series of compounds identified pirin as a molecular target for the compounds [32]. Pirin has also been shown to play a role in melanoma migration and senescence [33,34]. Building on our previous work showing that CCG-203971 inhibits melanoma metastasis [20] and that it can reverse *BRAF*-inhibitor resistance [35], here, we evaluated the pharmacological potential of CCG-222740, a more potent analog of CCG-203971, in combination with a MEK inhibitor (trametinib) in *NRAS*-mutant melanoma cells. In a panel of *NRAS* mutant human melanoma cell lines, we observed an association between the degree of activation of the Rho/MRTF pathway and intrinsic resistance of cells to trametinib-mediated apoptosis. Indeed, CCG-222740 was found to potentiate trametinib action in the subset of *NRAS* mutant melanoma cell lines that showed high activation of the Rho/MRTF pathway. In these cell lines, the combination of trametinib and CCG-222740 cooperatively induced apoptosis and reduced colony-forming potential.

## 2. Results

### 2.1. MRTF-Pathway Activation Correlates with Increased Trametinib Resistance

**Figure 1:**
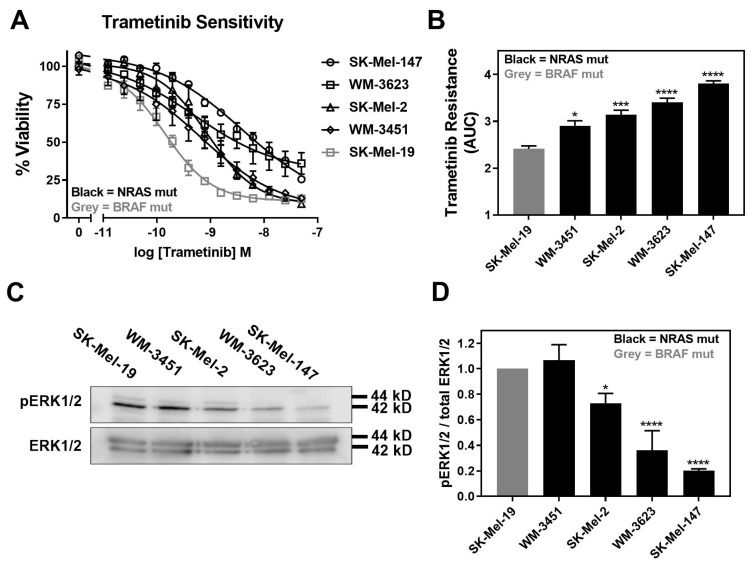
Increased trametinib resistance correlates with decreased pERK1/2. (**A**). Sensitivity to trametinib was determined by treating each cell line in 10% FBS with increasing concentrations of trametinib for 72 h. Cell viability was determined using Cell-TiterGLO^®^. Values are expressed as the fraction of luminescence over vehicle control for three independent experiments. (**B**). The area under the curve was plotted based on the concentration response curves generated in panel A using GraphPad Prism (n = 3, * *p* < 0.05, *** *p* < 0.01, **** *p* < 0.001 vs. SK-Mel-19). A greater area means less response to trametinib or increased trametinib resistance. (**C**). Western blot analysis of pERK1/2 across the cell line panel (n = 3). Each melanoma cell line was plated in 60 mm dishes in 10% FBS and harvested 24 h later. Image is a representative blot from three separate experiments. (**D**). Quantitative band density analysis was performed for each experiment comparing the intensity of pERK1/2 relative to total ERK. Results are expressed as the mean (±SEM) of triplicate experiments (n = 3, * *p* < 0.05, **** *p* < 0.001 vs. SK-Mel-19).

Trametinib is reported to induce apoptosis at varying levels and, overall, it is less potent/efficacious in *NRAS* mutant melanoma cell lines compared to *BRAF* mutant melanoma cell lines [5]. Therefore, we first determined the sensitivity of a panel of four *NRAS* mutant melanoma cell lines to inhibition of cell viability by trametinib treatment. In addition, we compared them to a *BRAF*^V600E^ mutant melanoma cell line, SK-Mel-19. The cell line panel included SK-Mel-147, SK-Mel-2, and WM-3451 cell lines, all of which harbor *NRAS*^Q61L^ mutations, and the WM-3451 cell line containing an *NRAS*^Q61K^ mutation. All *NRAS* mutant melanoma cell lines in our panel were less sensitive to trametinib treatment than the *BRAF* mutant SK-Mel-19 cell line (Figure 1A). The area under the curve (AUC) plotted based on the concentration–response curves demonstrated significantly greater trametinib resistance as well as significant variability among *NRAS* mutant cells compared to SK-Mel-19 (Figure 1B). Melanoma cells harboring *NRAS*^Q61^ mutations have previously been shown to have hyperactivation of the MAPK pathway [36]. Therefore, we quantified pERK1/2 from Western blots performed under basal conditions for each cell line (Figure 1C). We found that the levels of pERK1/2 varied substantially across the cell line panel (Figure 1D). This is not surprising given the poor correlation between patients’ mutation status and levels of pERK in melanoma tumors described clinically [37]. Decreasing amounts of pERK1/2 were strongly associated with trametinib resistance (Figure 1D).

Our recent findings indicated a role of the Rho/MRTF pathway in migration, invasion, and metastasis of aggressive human cutaneous melanoma [20], as well as in the acquired resistance of *BRAF* mutant melanoma cells to *BRAF* inhibitors [35]. Therefore, we hypothesized that the Rho/MRTF pathway might play a role in the intrinsic resistance of some of the *NRAS* mutant melanoma cells to trametinib-induced inhibition of cell viability. Upon activation of the Rho/MRTF pathway, myosin light chain (MLC) is phosphorylated, and G-actin polymerizes into F-actin stress fibers [38]. The depletion of G-actin during stress fiber formation results in nuclear translocation of MRTF where it regulates gene transcription, including an increase in cysteine-rich angiogenic inducer 61 (*CYR61*) [39]. We observed elevated levels of phosphorylated MLC (pMLC) in *NRAS* mutant cell lines compared to SK-Mel-19, with statistically significantly higher levels of pMLC in SK-Mel-2 cells (Figure S1). We stained the cells for F-actin and scored the images for stress fiber positive cells as described in the Methods section.

**Figure 2:**
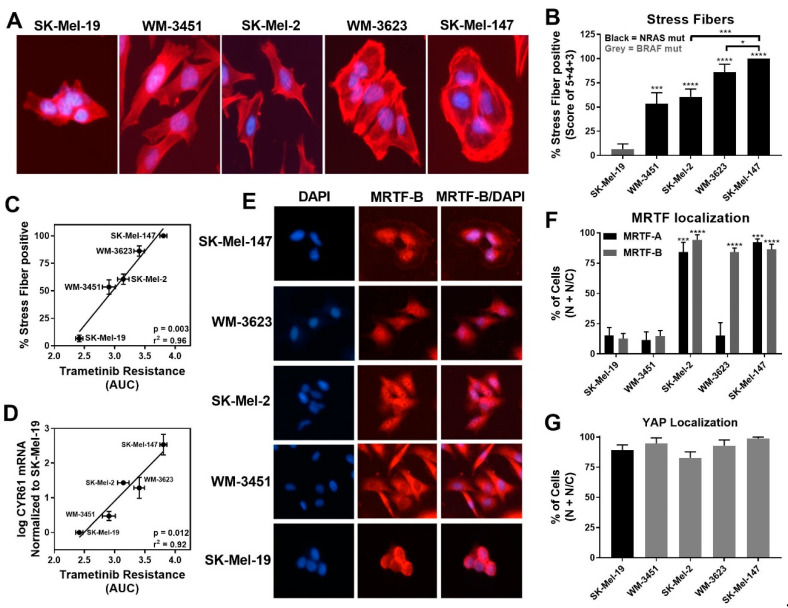
MRTF-pathway activation correlates with increased trametinib resistance. (**A**). Actin staining was assessed on the melanoma cell line panel using fluorophore-conjugated phalloidin toxin. (**B**). For stress fiber quantification, a score between 1 and 5 as described in Methods was utilized, and the percentage of cells scored as 3, 4, and 5 is presented as stress fiber-positive for each cell line (results are expressed as the mean ± SEM, n = 3, * *p* < 0.05, *** *p* < 0.005, **** *p* < 0.001 vs. SK-Mel-19 or SK-Mel-147). (**C**). The percentage stress fiber positivity correlates with trametinib resistance as assessed by the AUC for the trametinib concentration response curve (r^2^ = 0.96, *p* = 0.003, Pearson correlation coefficients, GraphPad Prism). (**D**). *CYR61* mRNA levels (n = 3) also correlate with trametinib resistance. (r^2^ = 0.92, *p* = 0.012, Pearson correlation coefficients, GraphPad Prism). (**E**). Immunolocalization of MRTF-B in melanoma cell lines. DAPI is used to stain nuclei. Images for MRTF-A in Figure S2. (**F**). Images were quantified by scoring individual cells as exclusively nuclear (N), exclusively cytosolic (C), or even distribution (N/C) of MRTF-A (black bars) or MRTF-B (gray bars). Counts are from three independent experiments with at least 100 cells scored for each cell line (n = 3, *** *p* < 0.005, **** *p* < 0.001 vs. SK-Mel-19). (**G**). Cellular localization of YAP using immunofluorescence was determined (n = 3, Figure S2B) and quantified as described for MRTF isoforms. No significant difference was observed across the melanoma cell lines.

While lower levels of pERK1/2 negatively correlated with trametinib sensitivity, we detected increased stress fiber formation in *NRAS* mutant cell lines compared to the *BRAF* mutant cell line SK-Mel-19. Among the *NRAS* mutant cell lines, SK-Mel-147 cells, which had the lowest basal pERK level, showed the strongest stress fiber positivity (Figure 2A,B). Quantitatively, the percentage of stress fiber positive cells showed strong positive correlation (r^2^ = 0.96, *p* = 0.003) to trametinib resistance (AUC, Figure 2C). This correlation was still strong when just comparing among *NRAS* mutant cell lines (r^2^ = 0.95, *p* = 0.024) Similarly, a significant correlation between the levels of *CYR61* mRNA levels and resistance to trametinib (AUC) was observed for all melanoma lines (r^2^ = 0.92, *p* = 0.012) and for all *NRAS* mutant lines (r^2^ = 0.74, *p* = 0.0003) (Figure 2D).

Since *CYR61* gene expression could be regulated upon activation of either the Rho/MRTF pathway via nuclear MRTF-A/B or the Hippo pathway via nuclear YAP, respectively [40], we next studied the localization of these transcription factors. Either MRTF-A or MRTF-B were strongly nuclear in three of the four *NRAS* mutant cell lines but not in WM-3451 or SK-Mel-19 cells (Figure 2E,F and Figure S2A). Interestingly, WM-3623 only had MRTF-B in the nucleus but not MRTF-A (see Discussion). Finally, we detected a high percentage of cells with nuclear YAP in our cell panel, including the *BRAF* mutant SK-Mel-19 cells, with no significant differences across the cell lines (Figure 2G and Figure S2B). Taken together, these results indicate that the Rho/MRTF pathway is activated in *NRAS* mutant melanoma cell lines having intrinsic resistance to trametinib-induced inhibition of cell proliferation.

### 2.2. An MRTF Pathway Inhibitor, CCG-222740, Synergizes with Trametinib to Reduce the Viability of NRAS Mutant Melanoma Cells

**Figure 3:**
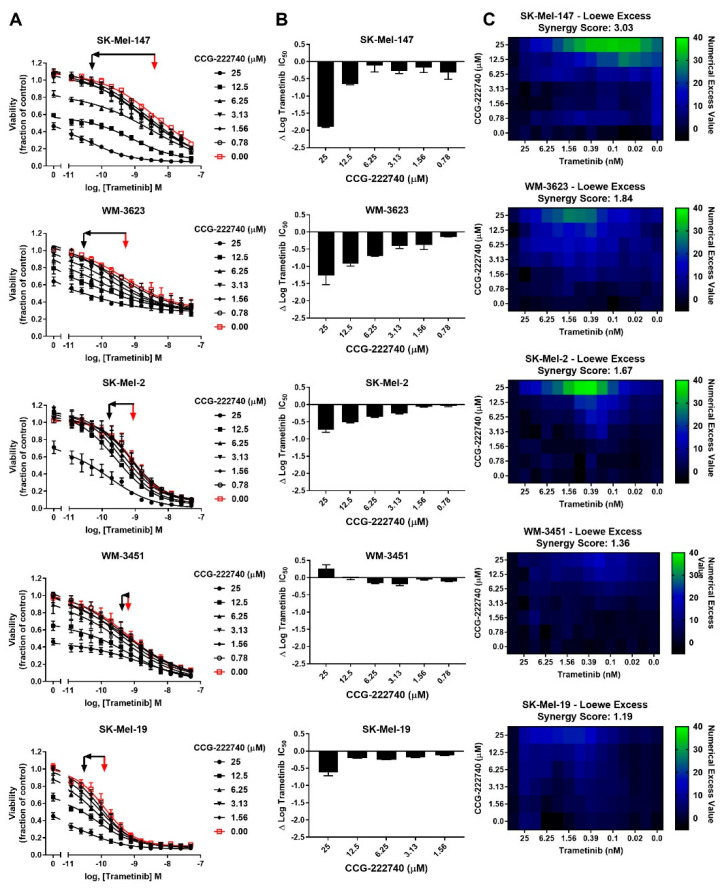
MRTF-pathway inhibition synergizes with trametinib to inhibit *NRAS* mutant melanoma cell viability. (**A**). Sensitivity to CCG-222740 and trametinib in combination was determined by treating each cell line with increasing concentrations of trametinib in the presence of a range of concentrations of CCG-222740. Cell viability at 72 h was determined using Cell-TiterGLO^®^. Viability is expressed as the fraction of luminescence over vehicle control. For SK-Mel-19 and SK-Mel-147, n = 4, for the other lines n = 3. The red arrow indicates the logIC50 of trametinib in the absence of CCG-222740, and the black arrow indicates the logIC50 of co-treatment conditions that displayed the greatest leftward shift in the trametinib concentration response curve (12.5 nM or 25 nM CCG-222740). (**B**). ∆logIC50 of trametinib is determined as the difference between the logIC50 of trametinib in the absence and at the indicated concentration of CCG-222740. (**C**). Loewe Excess was utilized as a metric of synergistic effects of the combination treatments. Loewe Excess was determined by detecting Numerical Excess Values (see Methods Section 4.2). Expressed values represent the mean of at 3–4 independent experiments.

Considering a potential role of Rho/MRTF pathway activation in the intrinsic resistance of *NRAS* mutant cell lines to trametinib treatment, we next sought to determine whether the Rho/MRTF-pathway inhibitor CCG-222740 could potentiate the effects of trametinib on viability in our *NRAS* mutant cell line panel (Figure 3A). Using cross-concentration response experiments, we observed that SK-Mel-147 cells demonstrated a significant leftward shift in log IC50, with a nearly 2 log-shift at the highest concentration of CCG-222740 utilized (Figure 3A,B). WM-3623 cells and SK-Mel-2 cells also showed potentiation of trametinib efficacy when trametinib was combined with CCG-222740 (see ∆log IC50 plots, Figure 3A,B). However, in WM-3451 cells, which show little Rho/MRTF activation, no potentiation of trametinib was observed when combined with CCG-222740 (Figure 3A,B). To determine if the observed leftward shifts in log IC50 of trametinib in combination with CCG-222740 represent a synergistic effect, we calculated the Loewe Excess to derive a synergy score (see Methods Section 4.2). Modest synergy was observed for the three *NRAS* mutant cell lines that had the greatest intrinsic trametinib resistance and the highest Rho/MRTF-pathway activity (Figure 3C). These results indicate that CCG-222740 synergistically acts with trametinib in *NRAS* mutant melanoma lines with intrinsic trametinib resistance.

### 2.3. CCG-222740 Disrupts Nuclear Localization of MRTF-A and MRTF-B But Not YAP

**Figure 4:**
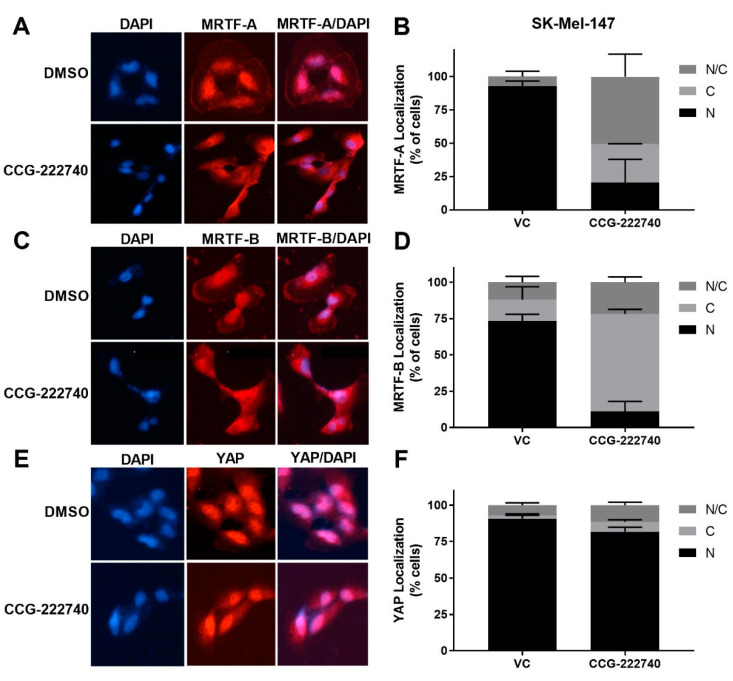
CCG-222740 disrupts nuclear localization of MRTF-A and MRTF-B but not YAP1. (**A**). Cellular localization of MRTF-A was determined in SK-Mel-147 cells by immunofluorescence following 24-h treatment with vehicle control (VC) 0.1% DMSO or 10 µM CCG-222740 in the presence of 10% FBS. (**B**). Quantification of MRTF-A cellular localization was determined by scoring individual cells as exclusively nuclear (N), cytosolic (C), or even distribution (N/C). Counts were determined from two independent experiments with at least 100 cells scored blindly for each condition (n = 2, >100 cells each determination). (**C**). Cellular localization of MRTF-B was determined in SK-Mel-147 cells as indicated above for MRTF-A. (**D**). Quantification of MRTF-B cellular localization was determined as indicated above for MRTF-A (n = 2, >100 cells each determination). (**E**). Counts are from cellular localization of YAP1 was determined in SK-Mel-147 cells as stated above for MRTF-A. (**F**). Quantification of YAP1 cellular localization was determined as indicated above for MRTF-A except that counts are from three independent experiments, n = 3, with at least 100 cells scored blindly for each condition.

As previously reported [20] for a structurally similar compound, CCG-203971, we found that CCG-222740 effectively reduced MRTF-A nuclear localization in a concentration-dependent manner in SK-Mel-147 cells (Figure 4A,B). It also inhibited the nuclear localization of MRTF-B (Figure 4C,D). However, no significant difference in the nuclear localization of YAP was observed following CCG-222740 treatment (Figure 4E,F). To gain insights into the CCG-222740-mediated gene transcription changes that might contribute to our observations, we undertook an RNA-Seq analysis of SK-Mel-147 cells treated with CCG-222740 for 24 h (Table S1). As expected, we found decreased mRNA levels of MRTF target genes *CYR61*, *ANKRD1*, *CRIM1* and *THBS1* upon treatment with CCG-222740 (Table S1), which was confirmed by qRT-PCR analysis (Figure S3). These results cement the importance of CCG-222740 as a negative regulator of the Rho/MRTF pathway in *NRAS* mutant melanoma.

### 2.4. Potentiation of Trametinib Action Is Specific to Rho/MRTF-Pathway Mechanisms

**Figure 5:**
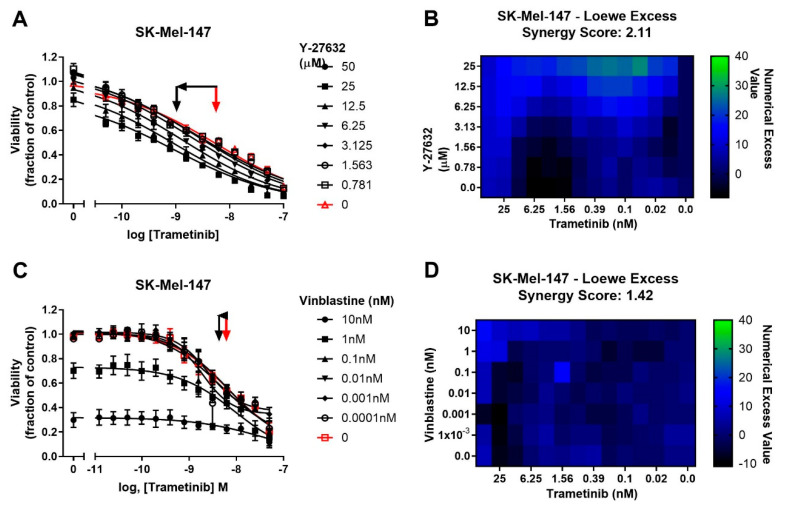
Potentiation of trametinib action is specific to the Rho/MRTF-pathway mechanisms. (**A**). Sensitivity to trametinib in combination with the ROCK inhibitor Y-27632. Combination treatments were determined in the same manner as described in Figure 3A.The reported values are expressed as the fraction of luminescence over vehicle control for three independent experiments. (**B**). Loewe Excess was calculated as a metric of synergistic effects of the combination treatments as described in Methods (Section 4.2). Values represent the mean of three independent experiments (n = 3). (**C**). Sensitivity to trametinib in combination with Vinblastine. Combination treatment was determined in the same manner as in Figure 5A. (**D**). Loewe Excess was determined as described in Methods (Section 4.2). Values represent the mean of three independent experiments (n = 3).

To determine whether potentiation of trametinib by CCG-222740 is specific to Rho/MRTF-pathway inhibition or merely a result of combination of trametinib with a cytotoxic agent, we first tested trametinib in combination with the microtubule assembly disrupting chemotherapeutic agent, vinblastine [41]. Vinblastine alone, when used at 1–10 nM, efficiently decreased SK-Mel-147 cell viability, yet when used in combination with trametinib, there was no significant shift in the log IC_50_ of trametinib (Figure 5A). Accordingly, a low synergy score of 1.42 was calculated for the combination of vinblastine and trametinib (Figure 5B). We next selected another agent to perturb Rho/MRTF signaling [42]. ROCK is a key component of the Rho/MRTF-pathway that acts upstream of MRTF activation. When Y-27632 was used in combination with trametinib in SK-Mel-147 cells, a significant, nearly one log shift in log IC_50_ was observed compared to trametinib alone (Figure 5C). A high synergy score of 2.11 was determined based on Loewe Excess analysis (Figure 5D). These results demonstrate a synergistic relationship between inhibitors of the Rho/MRTF-pathway and trametinib in suppressing *NRAS* mutant melanoma, whereas the effects of the non-targeted agent vinblastine and trametinib are additive.

### 2.5. Combination Treatment with Trametinib and CCG-222740 Inhibits Clonogenicity and Induces Apoptosis in SK-Mel-147 Cells

**Figure 6:**
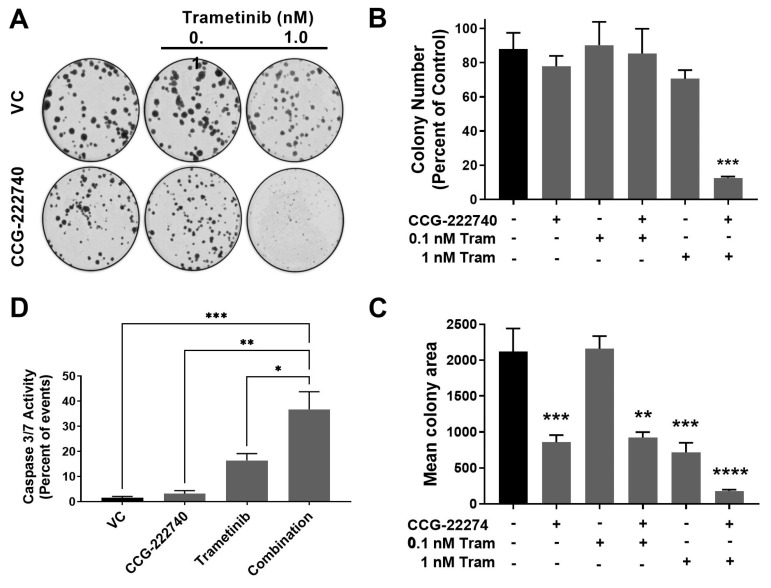
Combination treatment with trametinib and CCG-222740 inhibits clonogenicity and induces apoptosis in SK-Mel-147 cells. (**A**). SK-Mel-147 cells were seeded at low density (200 cells/well) into 6-well plates and simultaneously treated with DMSO control, 6 µM CCG-222740, 0.1 nM Trametinib, 1 nM Trametinib, or a combination of each. Representative clonogenic experiment is depicted. (**B**). Colony number was determined using ImageJ with a cutoff for colony size ≥ 50 pixels and circularity defined as 0.2–1.0. Results are expressed as the mean (±SEM) of triplicate experiments (n = 3, *** *p* < 0.005 vs. VC). (**C**). Mean colony area was determined using ImageJ, and the results are expressed as the mean (±SEM) of triplicate experiments (n = 3, ** *p* < 0.01, *** *p* < 0.005, **** *p* < 0.0001 vs. VC). (**D**). Caspase 3/7 activity was determined using flow cytometry 48 h after treatment with 10 µM CCG-222740 and 12.5 nM Trametinib alone or in combination and 0.2% DMSO VC. The combination showed a significant increase in caspase activation compared to all other conditions. At that low concentration of trametinib, the increase was not statistically significant. Results are expressed as the mean (±SEM) of triplicate experiments (n = 3, * *p* < 0.05, ** *p* < 0.01, *** *p* < 0.005 vs. VC, one-way ANOVA with an all-against-all Tukey’s multiple comparison test).

The viability of SK-Mel-147 *NRAS* oncogenic melanoma cells that show high resistance to MEK inhibitors was decreased by combined treatment with trametinib and CCG-222740. To further evaluate the functional effects of the combination treatment, we performed a colony formation assay to assess the clonogenicity of SK-Mel-147 cells in the presence of trametinib and CCG-222740 (Figure 6A–C). Treatment with CCG-222740 alone (6 µM) or trametinib alone (1 nM) had no significant effect on the number of colonies formed by SK-Mel-147 cells (Figure 6B). However, when cells were treated with the combination of 1 nM trametinib and 6 µM CCG-222740, colony formation was nearly eliminated (Figure 6B,C). When we evaluated mean colony area, we detected significant decreases across single agent and combination treatments except for 0.1 nM trametinib alone (Figure 6D). The greatest decrease in mean colony area was detected when cells were treated with 1 nM trametinib in combination with 6 µM CCG-222740 (Figure 6D). Thus, combination treatment with trametinib and CCG-222470 markedly inhibited clonogenicity of SK-Mel-147 cells.

Finally, we assessed Caspase3/7 activity as a measure of apoptosis induction. CCG-222740 alone (10 µM) did not affect basal apoptosis, as indicated by no change in caspase3/7 activity compared to control (Figure 6D). Low nanomolar trametinib increased caspase3/7 activity compared to untreated cells, although the effect was not statistically significant. However, when trametinib was used in combination with CCG-222740, a significant increase in caspase3/7 activity was observed in SK-Mel-147 cells vs. all other conditions (Figure 6D). These data provide evidence that SK-Mel-147 cells, which are highly resistant to trametinib-induced cell death, demonstrate a cooperative induction of apoptosis when the Rho/MRTF-pathway inhibitor CCG-222740 is combined with trametinib.

## 3. Discussion

Given the current lack of approved targeted therapies for *NRAS* mutant melanoma patients, MEK inhibitors offer a potential treatment option. However, intrinsic and acquired resistance to these inhibitors is a major hurdle. Clearly, there are factors beyond just the genomic diver mutations control melanoma growth and sensitivity to MAPK pathway inhibitors. Clinically, even with *NRAS* and *BRAF* mutations, the levels of pERK are highly variable as noted here and in clinical samples [37]. So, identifying the other factors that drive melanoma drug sensitivity is important. In a recent study, Najem et al. observed that MEK inhibition using pimasertib had only a limited effect on apoptosis in *NRAS*^Q61^ mutant melanoma cell lines [14]. They explained this by the systematic upregulation of microphthalmia-associated transcription factor (MITF), which, in turn, induces the expression of antiapoptotic Bcl-2 upon pimasertib treatment. Here, we show that a panel of four *NRAS*^Q61^ mutant melanoma cell lines had varying sensitivity to trametinib treatment and that the Rho/MRTF pathway was activated in the subset of *NRAS* mutant melanoma cell lines with high intrinsic resistance to trametinib-mediated cell growth inhibition.

Amplification of RhoA/C or MRTF-A/B or mutations in upstream activators of RhoA/C have been found in ≈30% cutaneous melanomas. Increased expression of RhoC or MRTF-A has been linked to aggressive disease and poor patient overall survival [20]. While the MRTF-SRF transcriptional axis plays a pro-metastatic role in cancers, upregulation of the Rho/MRTF pathway is also emerging as a drug resistance mechanism in different types of skin malignancies. Whitson et al. reported that activation of the Rho/MRTF pathway promoted resistance to a smoothened (SMO) inhibitor in basal cell carcinoma (BCC) [43]. Treatment with the MRTF pathway inhibitors CCG-1423 and CCG-203971 had considerable efficacy in treating resistant BCC in vivo, indicating a therapeutic potential of this pathway in drug-resistant malignancies. Additionally, we recently reported Rho/MRTF pathway activation in a subset of *BRAFBRAF*^V600^ mutant melanoma cell lines that are resistant to *BRAF*/MEK inhibitors [35]. Inhibition of the Rho/MRTF pathway via CCG-222740 re-sensitized the resistant cells to vemurafenib. Another recent report found activation of SRF-regulated gene transcription in Rac1^P29S^ mutant melanomas that was suppressed by a related compound CCG-257081 [44]. Here, we observed greater activation of the Rho/MRTF pathway in SK-Mel-147 and other *NRAS* mutant melanoma cells compared to the *BRAF* mutant SK-Mel-19 cells, as measured by stress fiber formation, nuclear localization of MRTF-A/B, and mRNA expression levels of the MRTF-target gene, *CYR61*. Importantly, Rho/MRTF pathway activation correlates strongly with intrinsic resistance to trametinib in these *NRAS* mutant cell lines. In light of the inverse relationship between pERK and Rho/MRTF activation, it is plausible that the Rho/MRTF pathway takes over for the MAPK pathway in driving cell proliferation and survival.

Considering the lack of success of therapies targeting *BRAF* and MEK in *NRAS* mutant melanoma patients [45], recent efforts have been focused on finding other therapeutic targets and the development of combination therapies. For example, inhibiting a novel *NRAS*-activating kinase (STK19) and combining MEK inhibitors with inhibitors of the MER receptor tyrosine kinase (MERTK), BET, and HDAC have been reported to block *NRAS* mutant melanoma growth in vitro and in vivo [15,16,17,45]. In this study, we demonstrate that the combination of a Rho/MRTF pathway inhibitor, CCG-222740, and the MEK inhibitor trametinib synergistically inhibits the viability of a subset of *NRAS* mutant melanoma cells and induced apoptosis in melanoma lines with a highly activated Rho/MRTF pathway. Similar results on cell viability were obtained when trametinib was used in combination with an inhibitor of ROCK, which is an important component of the Rho/MRTF pathway. Our results are in accordance with previous reports that demonstrated that a combination of MEK and ROCK inhibitors not only reduced *NRAS* mutant melanoma cell viability in vitro but also reduced tumor growth in vivo [12]. In SK-MEL-147 cells, which are the most trametinib-resistant cells in our panel, a low concentration of trametinib (12.5 nM) in combination of 10 µM CCG-222740 was able to induce apoptosis. This trametinib concentration is consistent with or lower than those in other in vitro studies involving synergistic combination of trametinib with other small molecule inhibitors [14,16,46]. For example, Vogel et al. reported a cooperative induction of apoptosis in SK-MEL-147 cells when a combination of 100 nM trametinib and 1 µM ROCK inhibitor GSK269962A was used [12]. Remarkably, in our studies, only 1 nM trametinib was needed along with 6 µM CCG-222740 to dramatically suppress the clonogenicity of the SK-Mel-147 cells. In addition, 12.5 nM trametinib and 10 µM CCG-222740 induced apoptosis in SK-Mel-147 cells. The use of such low MEK inhibitor concentrations is a significant benefit because severe dose-limiting toxicity is a concern with MEK inhibitors.

We observed that treatment with CCG-222740 disrupted the nuclear localization of MRTF in SK-Mel-147 cells, which was accompanied by the downregulation of MRTF target genes *CYR61*, *ANKRD1*, *CRIM1,* and *THBS1*. These genes in the MRTF–SRF axis can also be regulated by YAP, which is a transcription factor in the Hippo pathway. Using *CYR61* as a model target gene, Yu et al. demonstrated that the activation of both MRTF-A and YAP pathways and functional interactions between MRTF-A and YAP are required for the transcriptional control of RhoA-regulated genes in glioblastoma cells [47]. Foster et al. further showed that in cancer-associated fibroblasts, the expression of MRTF–SRF target genes and expression of YAP target genes are interdependent, even when only one of these pathways directly regulates the target gene [48]. They further found that the activation of a single pathway indirectly activates the other pathway in a manner dependent on cytoskeletal dynamics. Here, we found that YAP was localized in the nucleus of all the cell lines tested, irrespective of the *BRAF* or *NRAS* mutational status or sensitivity of cells to trametinib. Additionally, YAP remained localized in the nucleus even after the treatment with CCG-222740. We attempted to investigate the involvement of YAP and Hippo pathway in the potentiation of trametinib by CCG-222740 by using verteporfin, which is an inhibitor of YAP [49]. However, we were unable to obtain consistent and reproducible results in the cell viability assay when testing the effects of combination of verteporfin and trametinib (data not shown).

In addition to the effect of our compounds on Rho/MRTF pathway regulated gene transcription, they have recently been shown to bind directly to the cupin-family protein pirin [32]. Pirin has been reported to bind to and/or modulate multiple transcription factors and coregulators, including NFI/CTF1, HSF1, NFkB/p65, and Bcl3 [50,51,52]. We found that pirin overexpression or knock-down can modulate MRTF/SRF-regulated gene transcription [32]. The function of pirin in general, and specifically in melanoma, remains poorly defined. It is reported as either nuclear or cytoplasmic in different contexts [52,53]. In melanoma, its localization may be modulated by cell state [53]. In addition, a pirin-binding compound TphA can suppress melanoma cell migration [34] in a manner similar to that of our pirin-binding MRTF pathway inhibitors [20]. Another pirin-binding compound CCT251236 was found to be effective in an ovarian cancer xenograft model [51]. For all of these studies, it is not entirely clear whether the actions of these pirin-binding compounds are mediated through effects on Rho/MRTF mechanisms or perhaps through actions of pirin on other transcription mechanisms or even through non-pirin mechanisms. Further studies with pirin knockout mice would be valuable to assess these questions.

In conclusion, we report a role of the Rho/MRTF pathway in the intrinsic resistance of *NRAS* mutant melanoma cells to MEK inhibitor-induced cell death. There is a strong inverse correlation between measures of Rho/MRTF activation and sensitivity to trametinib. CCG-222740, a compound that inhibits Rho/MRTF-mediated gene transcription, markedly increased the efficacy of trametinib on *NRAS*-mutant melanoma cell lines. The combination of CCG-222740 and trametinib induced apoptosis and inhibited clonogenicity in those *NRAS* mutant melanoma cell lines that have increased Rho/MRTF activation and are strongly resistant to trametinib. These results warrant further in vivo studies with potential for clinical development of combinations of MEK inhibitors with Rho/MRTF pathway inhibitors.

## 4. Materials and Methods

### 4.1. Inhibitors

CCG-222740, previously reported by Hutchings et al., [30], was produced by the Vahltechich Medicinal Chemistry Core (Ann Arbor, MI, USA). CCG-222740 was dissolved in DMSO in 10 mM aliquots and stored at −20 °C. Trametinib (cat# S2673), Vinblastine (cat# S1248), Y-27632 (cat# S1049) were purchased from Selleck Chemicals (Houston, TX, USA), reconstituted in DMSO, and 10 mM aliquots were stored at −20 °C.

### 4.2. Cell Culture and Viability Assay

Human cutaneous melanoma cell lines WM-3451 and WM-3623 were purchased from The Wistar Institute (Philadelphia, PA, USA), SK-Mel-2 cells were purchased from ATCC (Manassas, VA, USA) and SK-Mel-19 and SK-Mel-147 were obtained from Dr. Maria Soengas at the University of Michigan and have been described previously [20]. Cells were cultured in DMEM (Life Technologies, Waltham, MA, USA) supplemented with 10% fetal bovine serum and 1× antibiotic-antimycotic solution (Life Technologies). Cells were expanded and frozen immediately prior to authentication and then thawed only two to three months before experiments. Short tandem repeat profiles were performed on SK-Mel-19 and SK-Mel-147 cell lines (Genewiz, South Plainfield, NJ, USA). The profiles obtained do not match any established published profiles, and we were unable to identify published profiles for SK-Mel-19 or SK-Mel-147. *NRAS* exon 3 and *BRAF* exon 15 were PCR amplified from genomic DNA and subjected Sanger sequencing (MSU Genomics Core). The mutation status of the cells used were *NRAS*^Q61L^ (SK-Mel-2, SK-Mel-147, WM-3451), *NRAS*^Q61K^ (WM-3623), *BRAF*^V600E^ (SK-Mel-19). For viability assays, 1000 cells in 20 µL of DMEM containing 10% FBS were seeded into 348-well white bottom plates. Four to six hours later, 10 µL of 4× compound was added along with an additional 10 µL of either 4× second compound or media. After 72 h, 20 µL of CellTiterGlo (Promega) was added to each well. The assay plate was centrifuged at 300× *g* for 3 min. Luminescence was measured using a BioTek Synergy Neo plate reader. Data were normalized to values obtained for the vehicle-treated cells. Data were plotted as average values of at least three independent experiments. Non-linear least square analysis was used to fit data to a 4-parameter log-[inhibitor] vs. response curve using GraphPad Prism versions 6–8 (GraphPad Software, La Jolla, CA, USA).

The Loewe Excess synergy score and graphical outputs were determined using the online Chalice software (cwr.horizondiscovery.com, accessed on 17 March 2021) which implements the Chou–Talalay method [54] for Loewe Excess calculations from concentration–response data. This method is currently available in the downloadable (https://sourceforge.net/projects/combenefit/, accessed on 17 March 2021) freeware Combenefit software [55].

### 4.3. Clonogenicity Assay

Two hundred cells in DMEM containing 10% FBS were seeded in 6-well dishes and simultaneously treated with vehicle or 6 µM CCG-222740 with or without 0.1 nM or 1 nM trametinib or with trametinib alone. Following five days of colony formation, fresh media and compound were added, and colonies were allowed to grow for an additional five days. Colonies were fixed and stained in 3.7% formaldehyde/0.5% crystal violet for 10 min at room temperature. Colony counts were quantified using ImageJ software with a cutoff for colony size ≥50 pixels and circularity defined as 0.2–1.0.

### 4.4. Immunofluorescence and F-Actin Staining

Cells (5 × 10^4^) were seeded into Falcon 8 Chamber Slides in 500 µL of DMEM containing 10% FBS and allowed to adhere overnight. Cells were fixed with 3.7% formaldehyde for 10 min at room temperature. Chambers were washed 3 times with 1× PBS for 5 min each. Cells were permeabilized with 0.25% Triton X-100 in PBS for 10 min followed by 3 5-min PBS washes. For stress fiber detection, rhodamine phalloidin (Cytoskeleton, Inc., Denver, CO, USA; cat# PHDR1) was used according to manufacturer’s recommendations. Stress Fibers were scored with slight modification from that described by Verderame et al. [56]. Score values followed the criteria: 5: >90% of cell area filled with thick cables; 4: At least two thick cables running under the nucleus and rest of area filled with fine cables; 3: No thick cables, but some fine cables present; 2: No cables visible in center area of cell; 1: No cable visible.

For MRTF-A staining, 10% donkey serum in PBS was used as a blocking solution for 30 min. For MRTF-B and YAP staining, 5% bovine serum albumin (BSA) in PBS was used as a blocking solution for 30 min. Primary antibodies for MRTF-A (Santa Cruz Biotechnology, Inc., Santa Cruz, CA, USA, cat# sc-21558), MRTF-B (Santa Cruz, cat# sc-98989), or YAP (Santa Cruz, cat# sc-15407) were diluted 1:100 in 1% BSA, 0.01% Triton X-100 in PBS and incubated at 4 °C overnight. Following 3 5-min PBS washes, secondary antibodies for MRTF-A (Alexa-Fluor^®^ 594 donkey anti-goat IgG antibody, Life Technologies, cat# A11058), for MRTF-B or YAP (Alexa-Fluor^®^ 594 goat anti-rabbit IgG antibody, Life Technologies, cat#A11037) were diluted 1:1000 in same primary antibody buffer and incubated 1 h at room temperature. Following 3 5-min PBS washes, cells were mounted (Prolong Gold antifade-reagent with DAPI, Invitrogen, Waltham, MA, USA, cat# P36935) and imaged on an EVOS FL Cell Imaging System (Life Technologies) at 40× magnification. All scoring of stress fibers and nuclear localization of transcription factors was done by an observer who was blinded to the identity of the sample.

### 4.5. Real-Time Quantitative PCR (qRT-PCR)

Cells (2 × 10^5^) were seeded into 6-well plates and left to adhere overnight; then, they were harvested the following day for *CYR61* mRNA analysis. Briefly, RNA was isolated using the RNeasy kit (Qiagen) following manufacturer’s directions. RNA (1 µg) was used as a template for synthesizing a cDNA utilizing Reverse Transcriptase Kit (Life Technologies, cat#4368814). Real-time PCR was conducted using SYBR Green Master Mix (Life Technologies, cat#4309155) using 4 µL of cDNA reaction on a Stratagene Mx3000P instrument (Agilent Technologies, Santa Clara, CA, USA), and cT values were analyzed relative to GAPDH expression. SK-Mel-19 cells, which have a low degree of Rho activation [20], were used to normalize relative *CYR61* mRNA levels for testing *NRAS* mutated cell lines. For *SRF* mRNA experiments, cells were treated with 2 nM trametinib and harvested at specified time points. DMSO was used to normalize relative expression. To assess the effects of CCG-222740 on *CYR61*, *CRIM1*, *THBS1,* and *ANKRD1* mRNA levels, 4.5 × 10^5^ cells were seeded into 6-well plates and left to adhere overnight. Then, cells were treated with 10 µM CCG-222740 for 24 h. RNA isolation and cDNA synthesis were performed as mentioned above. Real-time PCR was conducted using SYBR Green Master Mix and 2 µL of cDNA on the Stratagene Mx3000P instrument. cT values were normalized relative to GAPDH expression, and fold-change in mRNA expression upon CCG-222740 treatment was calculated relative to vehicle control (0.02% DMSO). Primer Sequences: *GAPDH* F: GGAGCGAGATCCCTCCAAAAT; *GAPDH* R: GGCTGTTGTCATACTTCTCATGG; *CYR61* F: CTCGCCTTAGTCGTCACCC′; *CYR61* R: CGCCGAAGTTGCATTCCAG; *SRF* F1: AGACGGGCATCATGAAGAAG; *SRF* R1: GATCATGGGCTGCAGTTTTC; *SRF* F2: CCTTCAGCAAGAGGAAGACG′; *SRF* R2: GATCATGGGCTGCAGTTTTC; CRIM1 F: GGTTCCTGTTGTGCTCTTGT; *CRIM1* R: TGCCAAGAATCAAGTTGCAGATAA; *THBS1* F: AGACTCCGCATCGCAAAGG; *THBS1* R: TCACCACGTTGTTGTCAAGGG; *ANKRD1* F: AGAACTGTGCTGGGAAGACG; *ANKRD1* R: GCCATGCCTTCAAAATGCCA.

### 4.6. Western Blot Analysis

Cells (8 × 10^5^) were seeded into 60-mm dishes. For both pERK1/2 and pMLC (Phospho-Myosin Light Chain 2, Thr18/Ser19) immunoblots, cells were harvested 24 h after seeding by direct lysis in 2× Laemmli buffer mixed with equal volume of RIPA buffer (10 mM Tris-Cl pH 8.0, 1 mM EDTA, 1% Triton X-100, 0.1% sodium deoxycholate, 0.1% SDS, 140 mM NaCl, protease/phosphatase inhibitor cocktail Thermo Fisher, Waltham, MA, USA, cat#88266). Samples were sonicated 2× for 5 s and heated for 5 min at 95 °C. Equal volumes of protein lysate (30 µL) for each cell line were resolved using 15% SDS-PAGE gels, transferred to PVDF membranes, and blocked in 5% BSA in Tween tris-buffered saline. Primary and secondary antibodies were diluted in 1% BSA and 0.01% Triton X-100 in PBS. Membranes were incubated in 1:1000 diluted primary antibodies pERK1/2 pT202/Y204 (Calbiochem, Burlington, MA, USA cat# KP26001), total ERK1/2 (Cell Signaling, Danvers, MA, USA, cat# 9102), pMLC (Cell Signaling, cat# 3674S), and GAPDH (Santa Cruz, cat#365062) overnight at 4 °C. HRP-conjugated anti-rabbit (Cell Signaling, cat# 7074P2), and HRP-conjugated anti-mouse (Cell Signaling, cat# 7076P2) antibodies were diluted 1:6000. Blots were developed using FemtoGlow™ Western chemiluminescent HRP substrate (Michigan Diagnostics, Royal Oak, MI, USA, cat# FWPS02). Bands were visualized and quantified using a Li-Cor Odyssey Fc scanner and quantified using Image Studio Lite Version 5.2. The full western blots were shown in Figures S4 and S5.

### 4.7. Caspase 3/7 Activity Assay

Cells (2 × 10^5^) were plated in 6-well plates in DMEM with 10% FBS and allowed to adhere overnight. Treatments were done the next day with either 10 µM CCG-222740 and 12.5 nM Trametinib alone or in combination and 0.2% DMSO was used as a vehicle control. CellEvent™ Caspase-3/7 Green Flow Cytometry Assay Kit (Invitrogen) was used according to the manufacturer’s protocol with modest modifications. Briefly, media and cells were harvested using a cell scraper 48 h after treatment started and centrifuged 300× *g* for 5 min. Cell pellets were washed with PBS and pelleted again. Cells were resuspended in 1 mL of PBS, and 150 µL of the cell solution was added to a V-bottom 96-well plate. The plate was centrifuged at 300× *g* for 5 min, and the PBS supernatant was removed. Single-stained compensation controls were included for each cell line for every experiment. Then, 25 µL of either FACs buffer (1% FBS in PBS) or CellEvent™ Caspase-3/7 Green Detection Reagent (diluted 1:1000 in FACS buffer) was added to appropriate wells, the plate was covered with film, and it was incubated at 37 °C at 5% CO_2_ for 25 min. The plate was removed, and 25 µL of either FACs buffer or SYTOX™ AADvanced™ dead cell stain (diluted 1:1000 in FACS buffer) was added to appropriate wells. The plate was covered with film and incubated at 37 °C for 5 min. Following incubation, 100 µL of FACs buffer was added to each well, and samples were transferred to 0.5 mL Eppendorf tubes in a 5% CO_2_ atmosphere. Fluorescence was detected using a C6 BD Accuri flow cytometer (BD Accuri, San Jose, CA, USA). Fluorescence was quantified using CFLow software (BD Accuri). Events (20,000) were detected in triplicate for all treatment groups and three independent experiments were conducted, and results were averaged.

### 4.8. RNA-Seq Sample Preparation and Data Processing

SK-Mel-147 cells were treated with or without 10 µM CCG-222740 or DMSO vehicle control for 24 h. Total cellular RNA was extracted from (two biological replicates per treatment condition) using the Qiagen, Hilden, Germany, RNeasy kit (#74104) according to the manufacturer’s protocol. RNA was eluted in nuclease-free H_2_O. RNA concentration was measured by Qubit and quality control was performed on an Agilent 2100 Bioanalyzer in the MSU Genomics Core. All RNA samples had a RIN score >8. Barcoded libraries were prepared using the Illumina TruSeq Stranded mRNA Library Preparation Kit on a Perkin Elmer Sciclone G3 robot following manufacturer’s recommendations. Completed libraries were QC’d and quantified using a combination of Qubit dsDNA HS and Caliper LabChipGX HS DNA assays. Libraries were pooled and run on two lanes, and sequencing was performed in a 1 × 50 bp single-end read format using HiSeq 4000 SBS reagents. Base calling was done by Illumina Real Time Analysis, RTA_v2.7.7 and output of RTA was demultiplexed and converted to FastQ format with Illumina Bcl2fastq v2.19.0. Sequencing was performed at a depth of >30 M reads/sample. Quality control was performed on the FastQ files using FastQC v0.11.5, and reads were trimmed using Trimmomatic v0.33. Reads were mapped using HISAT2 v2.1.0 and analyzed using HTSeq v0.6.1. Differential gene expression was calculated using edgeR. Raw RNA-Seq reads and processed HTSeq read counts are available on GEO under GSE134320.

### 4.9. Statistical Analysis

Comparisons among multiple samples used Ordinary One-way ANOVA in GraphPad Prism (v.6–8). A Dunnett’s post-test was used to compare all samples against a single control (e.g., SK-Mel-19 cells or untreated control cells) except in Figure 2B and Figure 6D where an all-against-all comparison was done with the Tukey’s post-test. Linear correlations and statistics were calculated using GraphPad Prism v.6–8. All studies had at least 3 biological replicates. *p* values of <0.05 were considered significant.

## 5. Conclusions

Melanoma tumors with mutations in *NRAS*, the second-most commonly mutated oncogenic driver in melanoma, have limited therapeutic targets. Blocking MEK, which is downstream of *NRAS*, has modest effects on these cancers, but MEK inhibitors alone do not provide clear clinical efficacy. In this study, we find that activation of the RhoA/C pathway and MRTF, a transcriptional regulator downstream of Rho and actin-cytoskeletal rearrangements, has a strong inverse correlation with response to trametinib. Furthermore, inhibitors of Rho/MRTF-regulated gene transcription, CCG-222740 and Rho-kinase inhibitor, markedly enhance the sensitivity of *NRAS* mutant melanoma cells to the MEK inhibitor trametinib. The combination of CCG-222740 with low concentrations of trametinib is able to suppress clonogenicity and induce the apoptosis of a highly aggressive *NRAS* mutant melanoma. Combination therapy with drugs able to inhibit Rho or MRTF signaling with existing MEK inhibitors may prove to be a valuable approach to this challenging cancer.

## Data Availability

RNA-seq data defining the effect of CCG-222740 on gene expression in SK-Mel-147 cells are available at the GEO repository under accession number GSE134320. Other primary data are shown in Tables S2 and S3 or are available from the corresponding author upon request.

## Supplementary Materials

The following are available online at https://www.mdpi.com/article/10.3390/cancers13092012/s1, Figure S1: Expression of phospho-myosin light chain (pMLC) in melanoma cell lines, Figure S2: Localization of MRTF-A and YAP in melanoma cell lines., Figure S3: Effect of CCG-222740 on expression of MRTF-target genes in SK-Mel-147 cells, Figure S4: Full blots for ERK and pERK supporting Figure 1C,D, Figure S5: Full blot for pMLC and GAPDH supporting Figure S1, Table S1: RNA-Seq analysis of differential gene expression in SK-Mel-147 cells upon 24-h treatment with CCG-222740, Table S2: Intensities for ERK and pERK supporting Figure 1C,D, Table S3: Intensities ratios for pMLC/GAPDH vs. SK-Mel-19 supporting Figure S1.
