# Supplementary material for: Inhibition of the Myocardin-Related Transcription Factor Pathway Increases Efficacy of Trametinib in NRAS-Mutant Melanoma Cell Lines
Source: Cancers (Basel). 2021 Apr 22;13(9):2012. doi: 10.3390/cancers13092012 (PMC8122681; doi:10.3390/cancers13092012)
Supplement: Supplementary file 1 [file cancers-13-02012-s001.zip › cancers-1180979-SI.pdf]

Supplementary Materials

Inhibition of the Myocardin-Related Transcription Factor Pathway Increases Efficacy of Trametinib in *NRAS*-Mutant Melanoma Cell Lines

Kathryn M. Appleton, Charuta C. Palsuledesai, Sean A. Misek, Maja Blake, Joseph Zagorski, Kathleen A. Gallo, Thomas S. Dexheimer and Richard R. Neubig

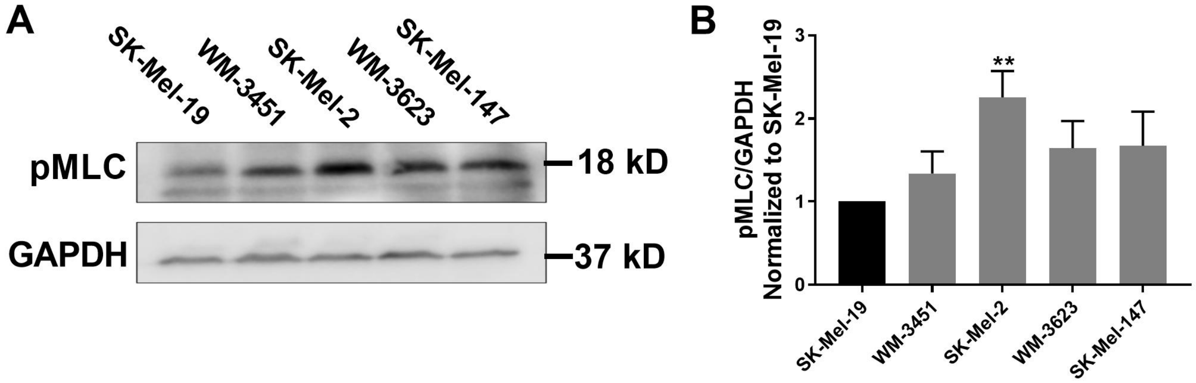

Supplementary Figure S1. **Expression of phospho-myosin light chain (pMLC) in melanoma cell lines. A.** Immunoblots were conducted across the melanoma cell line panel to detect pMLC or GAPDH as a protein loading control. **B.** Quantitative band density analysis was performed for each experiment comparing the intensity of pMLC relative to control GAPDH then normalized to the value for SK-Mel-19 from the same experiment. Results are expressed as the mean (±SEM) of five experiments (n=5, **p<0.01 vs. SK-Mel-19, One-way ANOVA, with Dunnett's post-test).

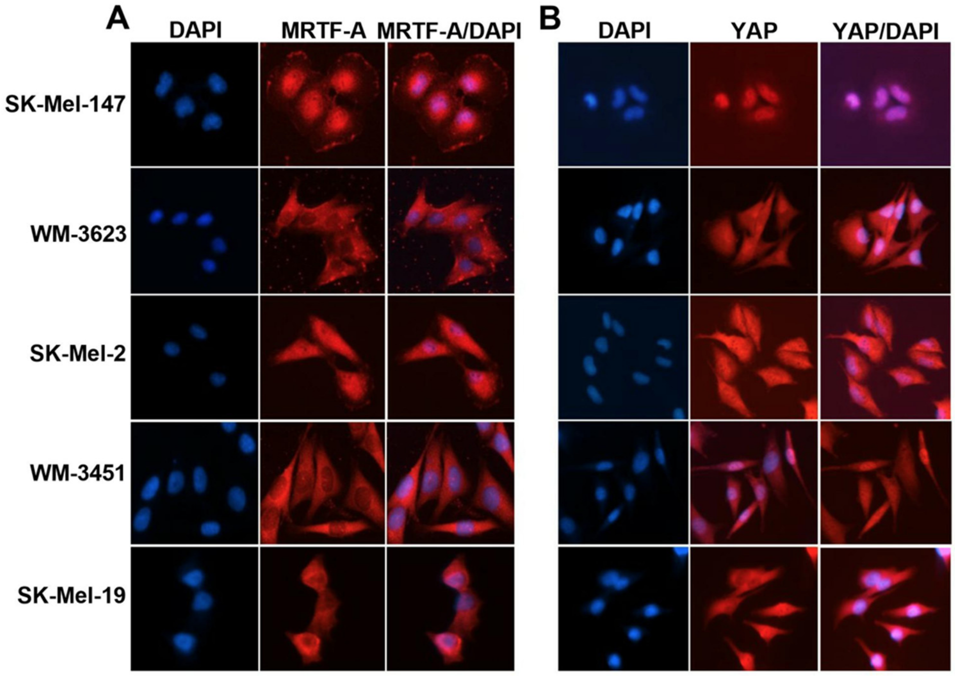

Supplementary Figure S2. **Localization of MRTF-A and YAP in melanoma cell lines. A.** Representative images of the cellular localization of MRTF-A using immunofluorescence in melanoma cell lines (10% FBS). **B.** Representative images of the cellular localization of YAP using immunofluorescence in melanoma cell lines (10% FBS).

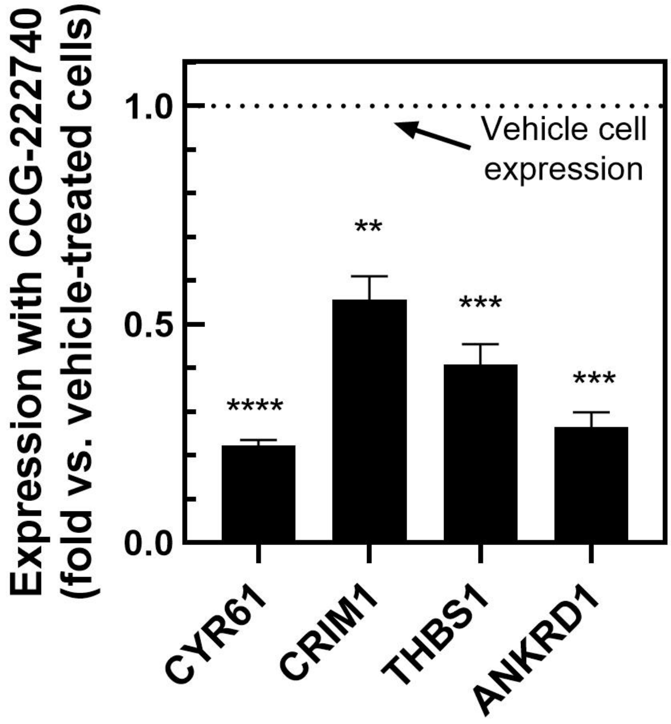

Supplementary Figure S3. **Effect of CCG-222740 on expression of MRTF-target genes in SK-Mel-147 cells.** CCG-2222740 treatment reduces expression of MRTF target genes *CYR61, CRIM1, THBS1* and *ANKRD1*. SK-Mel-147 cells were treated for 24 hours with 10 μM CCG-222740 or DMSO (0.02%) before isolation of RNA for qPCR analysis. Expression of target genes were normalized against *GAPDH* and results are expressed as the mean (±SEM) of triplicate experiments (n=3, *, P < 0.01, **, P < 0.001, ***, P < 0.0001, ****, P < 0.00001 vs. DMSO control).

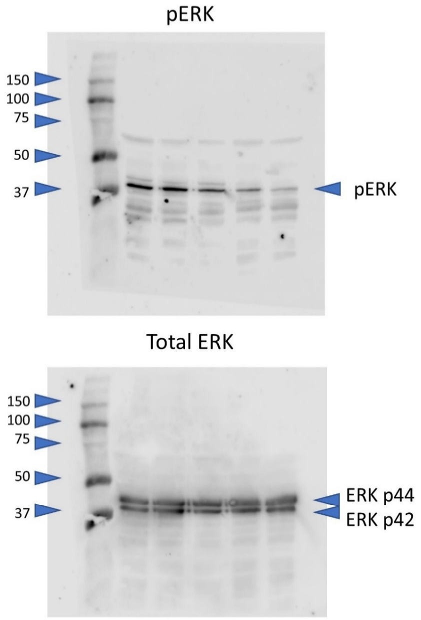

Supplementary Figure S4 Full blots for ERK and pERK supporting Figure 1C & D

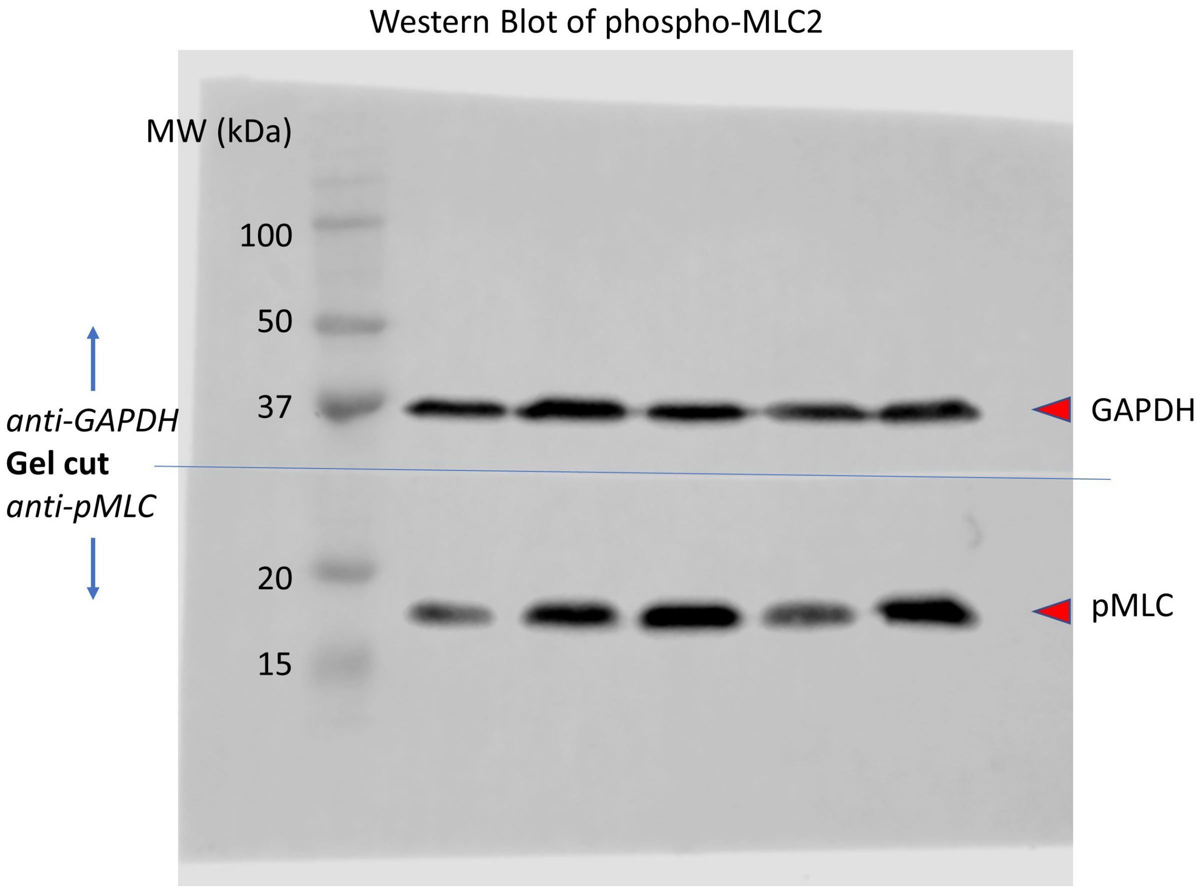

Supplementary Figure S5 Full blot for pMLC and GAPDH supporting Figure S1

Supplementary Table S1. RNA-Seq analysis of differential gene expression in SK-Mel-147 cells upon 24-hour treatment with CCG-222740. Effects of 10 mM CCG-222740 in SK-Mel-147 cells was compared to untreated cells. See Methods section 4.8 of main manuscript for experimental details. Negative logFC values indicate genes downregulated upon CCG-222740 treatment, and positive values indicate genes upregulated upon CCG-222740 treatment. Raw RNA-Seq reads and processed HTSeq read counts are available on GEO under GSE134320.

Ensembl ID	Approved Symbol	logFC	logCPM	PValue	FDR
**ENSG00000168003**	*SLC3A2*	1.740277527	8.507639444	1.21E-14	7.71E-10
**ENSG00000115380**	*EFEMP1*	-1.706565216	3.530197807	9.55E-11	3.04E-06
**ENSG00000091986**	*CCDC80*	-1.495390124	4.347019002	4.67E-09	9.92E-05
**ENSG00000181449**	*SOX2*	2.140598107	0.239972409	3.44E-08	0.000547402
**ENSG00000104419**	*NDRG1*	1.363870834	5.978803232	4.66E-08	0.000586459
**ENSG00000148677**	*ANKRD1*	-1.397055415	4.627081746	5.59E-08	0.000586459
**ENSG00000150938**	*CRIM1*	-1.308149346	6.808500344	6.45E-08	0.000586459
**ENSG00000224773**	*HSPA8P7*	-1.98036841	2.305467038	1.86E-07	0.001477206
**ENSG00000137801**	*THBS1*	-1.185139541	10.08725598	2.85E-07	0.00201518
**ENSG00000196878**	*LAMB3*	1.137023364	7.353579658	9.04E-07	0.005469966
**ENSG00000213820**	*RPL13P2*	-2.378109738	2.097258073	9.45E-07	0.005469966
**ENSG00000128564**	*VGF*	1.413051246	1.555022873	1.3E-06	0.006602879
**ENSG00000154734**	*ADAMTS1*	-1.102716883	6.357359483	1.35E-06	0.006602879
**ENSG00000236105**	*PRELID3BP10*	-2.521855225	-0.667607432	1.89E-06	0.008593398
**ENSG00000231445**	*TIMM8AP1*	-2.03756353	-0.277981649	2.78E-06	0.01016252
**ENSG00000129757**	*CDKN1C*	1.19260645	3.011058803	2.85E-06	0.01016252
**ENSG00000142871**	*CYR61*	-1.036485735	9.347827715	2.87E-06	0.01016252
**ENSG00000092969**	*TGFB2*	-1.146679925	4.714269799	3.1E-06	0.010396226
**ENSG00000052841**	*TTC17*	1.075378747	6.169282608	5.58E-06	0.017771644
**ENSG00000078401**	*EDN1*	-1.202295537	3.121136369	5.94E-06	0.018013191
**ENSG00000052802**	*MSMO1*	1.079766996	5.379290384	7.15E-06	0.020689773
**ENSG00000138798**	*EGF*	-1.282835697	2.550065363	7.79E-06	0.021564115
**ENSG00000170439**	*METTL7B*	1.152224231	2.41811322	1.06E-05	0.028090638
**ENSG00000114251**	*WNT5A*	-1.050718673	5.271773994	1.39E-05	0.032740269
**ENSG00000232480**	*TGFB2-AS1*	-1.851166895	0.032554217	1.4E-05	0.032740269
**ENSG00000168028**	*RPSA*	-1.157332873	6.684989777	1.44E-05	0.032740269
**ENSG00000145632**	*PLK2*	-0.9729373	7.098792127	1.44E-05	0.032740269
**ENSG00000151892**	*GFRA1*	-1.241962153	1.757886426	1.67E-05	0.035350063
**ENSG00000170523**	*KRT83*	-1.706326252	0.010133492	2.25E-05	0.045127787
**ENSG00000214274**	*ANG*	1.837466378	-0.700807455	2.27E-05	0.045127787

Supplementary Table S2 Intensities for ERK and pERK supporting Figure 1C & D
Individual band ratios from Figure 1 – three independent experiments

	SK-Mel-19	WM-3451	SK-Mel-2	WM-3623	SK-Mel-147
**Experiment 1**					
pERK	4.22	4.38	2.92	1.64	1.09
ERK1/2	7.96	8.8	8.36	9.21	9.77
pERK/ERK ratio	0.530	0.498	0.349	0.178	0.112
**norm to SK-Mel-19**	**1.000**	**0.939**	**0.659**	**0.336**	**0.210**
**Experiment 2**					
pERK	4.85	4.86	3.36	2.73	1.18
ERK1/2	28.6	24.3	27.5	30.6	33.2
pERK/ERK ratio	0.170	0.200	0.122	0.089	0.036
norm to SK-Mel-19	***1.000***	***1.179***	***0.720***	***0.526***	***0.210***
**Experiment 3**					
pERK	4.92	5.21	4.44	1.86	1.48
ERK1/2	12.4	12.1	13.8	20.8	20.4
pERK/ERK ratio	0.397	0.431	0.322	0.089	0.073
norm to SK-Mel-19	***1.000***	***1.085***	***0.811***	***0.225***	***0.183***

Supplementary Table 3 Intensities ratios for pMLC/GAPDH vs SK-Mel-19 supporting Figure S1

Individual band ratios from Figure S1

	SK-Mel-19	WM-3451	SK-Mel-2	WM-3623	SK-Mel-147
GAPDH	1.07	1.26	1.06	1.27	1.06
pMLC	0.97	1.11	1.89	1.36	1.49
pMLC/GAPDH	0.902	0.881	1.783	1.071	1.406
Norm to SK-Mel-19*	1*	0.977	1.977	1.187	1.559

Normalized band ratios from 5 independent experiments

SK-Mel-19	WM-3451	SK-Mel-2	WM-3623	SK-Mel-147
1	1.242	2.092	1.271	1.880
1	1.143	2.009	2.344	0.765
1	2.385	3.488	2.510	3.116
1	0.954	1.721	0.917	1.047
1*	0.977	1.977	1.187	1.559

* Data from blot in Figure S1
